# Association between metabolic syndrome and venous thromboembolism after total joint arthroplasty: a meta-analysis of cohort studies

**DOI:** 10.1186/s13018-020-02097-4

**Published:** 2020-11-30

**Authors:** Yipei Yang, Ziyue Li, Haifeng Liang, Jing Tian

**Affiliations:** 1 Department of Orthopedic and Joint Surgery, Zhu Jiang Hospital, Southern Medical University, No. 253 Middle Gongye Dadao, Haizhu District, Guangzhou, 510000 China; 2 Department of Doppler Ultrasonic Department, Shen Zhen Hospital, Southern Medical University, Shenzhen, 518000 China

**Keywords:** Metabolic syndrome, Total knee arthroplasty, Total hip arthroplasty, Venous thromboembolism, Meta-analysis

## Abstract

**Objective:**

Metabolic syndrome (MetS) has been associated with hypercoagulative status. However, previous studies evaluating the association between MetS and incidence of venous thromboembolism (VTE) after total joint arthroplasty (TJA) showed inconsistent results. We performed a meta-analysis to evaluate the influence of MetS on the risk of VTE following TJA.

**Methods:**

Cohort studies were identified by the search of PubMed, Embase, and the Cochrane’s Library databases. A random-effect model was used if considerable heterogeneity was detected; otherwise, a fixed-effect model was used. Subgroup analyses according to the category of VTE, definition of MetS, category of procedure, and follow-up durations were performed.

**Results:**

Seven cohort studies with 1,341,457 patients that underwent TJA were included, with 118,060 MetS patients (8.8%) at baseline. With a follow-up duration up to 3 months after surgery, 9788 patients had VTE. Pooled results with a random-effect model showed that MetS was not associated with increased overall VTE after TJA (adjusted risk ratio [RR] = 1.24, 95% confidence interval [CI] 0.89 ~ 1.72, *p* = 0.20; *I*^2^ = 69%). The results were not significantly affected by the diagnostic criteria of MetS, category of the procedure, and follow-up durations. Subgroup analyses showed that MetS was not associated with an increased the risk of pulmonary embolism ([PE], RR 1.06, 95% CI 0.37 ~ 3.02, *p* = 0.91), but an increased risk of deep vein thrombosis (DVT) after TJA (RR 3.38, 95% CI 1.83 ~ 6.24, *p* < 0.001).

**Conclusions:**

Current evidence from observational studies suggests MetS might be associated with an increased risk of DVT but not PE after TJA.

## Introduction

Patients undergoing total joint arthroplasty (TJA), including total knee arthroplasty (TKA) and total hip arthroplasty (THA), are at a higher risk for the development of venous thromboembolism (VTE) [1–3]. Despite the routine use of prophylactic measures against thrombosis in these patients, the incidences of VTE, including pulmonary embolism (PE) and deep vein thrombosis (DVT) remains high [4]. A previous systematic review including 44,844 cases from 47 studies showed that in patients receiving recommended prophylaxis, the pooled rates of symptomatic postoperative VTE before hospital discharge were 1.09% for patients undergoing TKA and 0.53% for those undergoing THA [5]. Moreover, VTE has become an important cause of morbidity and mortality in patients after TJA [6]. Identification of risk factors for VTE in patients that received TJA is clinically important [6].

Metabolic syndrome (MetS) refers to a cluster of metabolic abnormalities including abdominal adiposity, insulin resistance, hyperglycemia, hypertension, and dyslipidemia [7, 8]. Accumulating evidence suggests that patients MetS are characterized by hypercoagulative status [9–13]. Indeed, MetS has been associated with a 2-fold increase in arterial thrombotic diseases, such as coronary artery disease and stroke [14]. Moreover, a previous individual-patient data-based meta-analysis showed that MetS is associated with an increased risk of unprovoked VTE in general population [15]. Since MetS is prevalent in patients undergoing TJA, accumulating studies have evaluated the association between MetS and risk of VTE in these patients [16–22]. However, the results of these studies are inconsistent. Some of them suggested that MetS may be a risk factor for VTE after TJA [16, 19, 21], while other studies did not [17, 18, 20, 22]. Therefore, we aimed to perform a meta-analysis to evaluate the association between MetS and risk of VTE in patients after TJA. The influences of categories of VTE, definitions of MetS, and types of procedures on the association were also analyzed.

## Methods

The meta-analysis was designed and performed in accordance with the MOOSE (Meta-analysis of Observational Studies in Epidemiology) [23] and Cochrane’s Handbook [24] guidelines.

### Literature search

Electronic databases of PubMed, Embase, and the Cochrane’s Library were systematically searched using the combination of the following terms: [1] “metabolic syndrome” OR “insulin resistance syndrome” OR “syndrome X” [2]; “hip” OR “knee”; and [3] “arthroplasty” OR “replacement” OR “surgery” OR “operation”. We used this extensive search strategy to avoid missing potential studies. The search was limited to human studies published in English or Chinese. The reference lists of original and review articles were also analyzed manually. The final literature search was performed on April 2, 2020.

### Study selection

Studies were included if they met the following criteria: [1] published as full-length article, [2] designed as cohort studies, [3] included patients that received TKA or THA, [4] MetS was identified as exposure of interest at baseline, [5] documented the incidence of any VTE events (overall VTE, PE, or DVT) in patients with and without MetS, and [6] reported the adjusted risk ratios (RRs, at least adjusted for age and sex) and their corresponding 95% confidence intervals (CIs) for the incidence of VTE in patients with and without MetS. Definitions of MetS were consistent with what was applied in the original studies. Reviews, editorials, preclinical studies, and non-cohort studies were excluded.

### Data extracting and quality evaluation

Literature search, data extraction, and study quality assessment were independently performed by two authors according to the predefined inclusion criteria. If inconsistencies occurred, discussion with the corresponding author was suggested to resolve the disagreement. The following data were extracted: [1] name of the first author, publication year, study location, and study design [2]; characteristics and numbers of patients that received TKA or THA, criteria for the diagnosis of MetS, prevalence of MetS, and follow-up period; and [3] number of cases with any VTE events during follow-up, VTE prophylactic strategies, methods for the validation of the outcome, and variables adjusted. The quality of each study was evaluated using the Newcastle-Ottawa Scale (NOS) [25]. This scale ranges from 1 to 9 stars and judges the quality of each study regarding three aspects: the selection of the study groups, the comparability of the groups, and the ascertainment of the outcome of interest.

### Statistical analyses

The association between MetS and VTE in patients after TKA or THA was measured by RRs. To stabilize its variance and normalized the distribution, RR data and its corresponding stand error (SE) from each study was logarithmically transformed [24]. The Cochrane’s *Q* test was performed to evaluate the heterogeneity among the include cohort studies [24, 26], and the *I*^2^ statistic was also calculated. A significant heterogeneity was considered if *I*^2^ > 50%. A random effect model was used to pool the results if significant heterogeneity was detected; otherwise, a fixed-effect model was used. Sensitivity analysis by omitting one study at a time was performed to evaluate the stability of the results [24]. To evaluate the influences of categories of VTE, definitions of MetS, types of procedures, and follow-up durations on the association, subgroup analyses were performed. Potential publication bias was assessed by visual inspection of the symmetry of the funnel plots, complemented with the Egger regression test [27]. *P* < 0.05 was considered as statistically significant. The RevMan (Version 5.1; Cochrane Collaboration, Oxford, UK) and STATA software were used for the statistics.

## Results

### Literature search

**Fig. 1:**
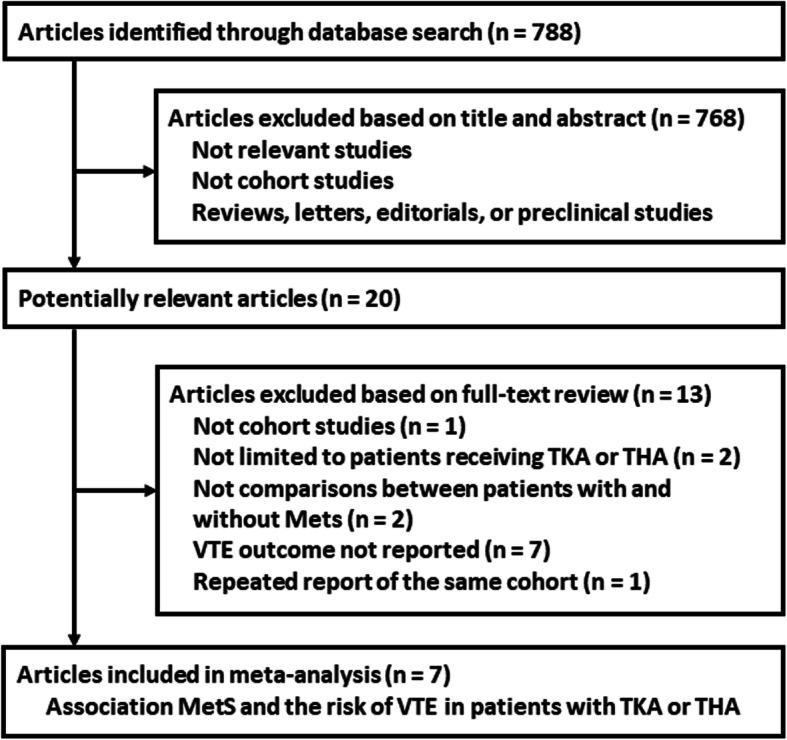
The flowchart of database search and study inclusion

The flowchart of the database search was shown in Fig. 1. Briefly, 788 studies were obtained from the database search, and 768 of them were excluded primarily due to the irrelevance to the purpose of the study. For the remaining 20 potential relevant studies that underwent full-text review, 13 were further excluded for the reasons listed in Fig. 1. Finally, seven cohort studies were included [16–22].

### Study characteristics and quality

Characteristics of the included studies were summarized in Table 1. Overall, seven cohort studies with 1,341,457 patients that underwent TJA were included [16–22]. These studies were performed in the USA, Canada, and China and published between 2009 and 2018. One of them was a prospective cohort study [16], and the others were retrospective cohorts [17–22]. Since two studies reported the incidence of VTE events in patients received TKA and THA separately, these datasets were independently included. Therefore, nine datasets were available for the meta-analysis, and 118,060 patients (8.8%) were diagnosed as MetS at baseline according to the World Health Organization (WHO) [16, 20, 21] or the National Cholesterol Education Program Expert Panel and Adult Treatment Panel III (NCEP-ATP III) criteria [17–19, 22]. The follow-up duration varied between within hospitalization and 3 months after surgeries, and 9788 patients had VTE during follow-up. Strategies for VTE prophylaxis were reported in three studies [16, 19, 21], and anticoagulants of warfarin, rivaroxaban, or low-molecular-weight heparin were used. The methods for VTE assessment were also reported in three studies [16, 19, 21], which involved the application of Doppler ultrasonography, venography, and CT and/or lung VQ scan. Potential confounding factors, including age, sex, body mass index (BMI), smoking, and comorbidities, were adjusted to a varying degree in the included studies. The qualities of the included follow-up studies were generally good, with the NOS ranging from 6 ~ 8 (Table 2).

**Table 1:** Characteristics of the included cohort studies

Study	Country	Design	Patient characteristics	Sample size	Mean age years	Male (%)	MetS diagnosis	MetS at baseline, *n* (%)	Follow-up duration	Prophylactic methods	Outcomes reported	Outcome validation	Variables adjusted
Gandhi 2009 [16]	Canada	PC	Patients received unilateral TKA	1460	66.5	35.9	WHO	135 (9.2)	3 months	LMWH	Symptomatic DVT (65)	DUS in systematic patients	Age, sex, education, BMI, and CCI
Dy 2011 [17]	The US	RC	Patients received TKA or THA	16317	64.8	39.9	NCEP-ATP III	1093 (6.7)	3 months	NR	VTE (148)	ICD-9 codes	Age, sex, procedure type, and BMI
Valle 2012-TKA [18]	The US	RC	Patients received TKA	806672	66.9	36.1	NCEP-ATP III	82852 (10.3)	In-hospital	NR	VTE (6476)	ICD-9 codes	Age, sex, race, admission type, comorbidities, and hospital level
Valle 2012-THA [18]	The US	RC	Patients received THA	406265	65.6	42.5	NCEP-ATP III	24269 (6.0)	In-hospital	NR	VTE (1986)	ICD-9 codes	Age, sex, race, admission type, comorbidities, and hospital level
Mraovic 2013 [19]	The US	RC	Patients received TKA or THA	7282	70.2	44.0	NCEP-ATP III	958 (13.2)	In-hospital	Warfarin to achieve INR: 1.5 ~ 2.0	Symptomatic PE (107)	CT and/or lung VQ scan in systematic patients	Age, sex, procedure type, BMI, and comorbidities
Song 2016-TKA [21]	China	RC	Patients received TKA	560	67.2	18.0	WHO	45 (8.0)	1 month	Rivaroxaban or LMWH	Symptomatic DVT (25)	Routine venography	Age, sex, comorbidities, and smoking
Song 2016-THA [21]	China	RC	Patients received THA	993	63.4	37.3	WHO	34 (3.4)	1 month	Rivaroxaban or LMWH	Symptomatic DVT (53)	Routine venography	Age, sex, comorbidities, and smoking
Edelstein 2016 [20]	The US	RC	Patients received TKA or THA	1462	NR	36.5	WHO	237 (16.2)	1 month	NR	PE (33)	Medical insurance data	Age, sex, BMI, and comorbidities
Cichos 2018 [22]	The US	RC	Patients with hip fracture received THA	100446	77.6	31.2	NCEP-ATP III	8437 (8.4)	In-hospital	NR	VTE (904)	ICD-9 codes	Age, sex, race, payer status and comorbidities

*MetS* metabolic syndrome, *TKA* total knee arthroplasty, *THA* total hip arthroplasty, *US* United States, *PC* prospective cohort, *RC* retrospective cohort, *WHO* World Health Organization, *NCEP-ATP III* the National Cholesterol Education Program Expert Panel and Adult Treatment Panel III, *PE* pulmonary embolism, *DVT* deep vein thrombosis, *VTE* venous thromboembolism, *DUS* Doppler ultrasonography, *ICD-9* the 9th revision of International Classification of Diseases, *CT* computed tomography, *VQ* ventilation/perfusion, *BMI* body mass index, *CCI* Charlson Comorbidity Index, *NR* not reported, *INR* international normalized ratio, *LMWH* low-molecular-weight heparin

**Table 2:** Details of the study quality evaluation via the Newcastle-Ottawa Scale

Study	Representativeness of the exposed cohort	Selection of the non-exposed cohort	Ascertainment of exposure	Outcome not present at baseline	Control for age and sex	Control for other confounding factors	Assessment of outcome	Enough long follow-up duration	Adequacy of follow-up of cohorts	Total
Gandhi 2009 [16]	1	1	1	0	1	1	1	1	1	8
Dy 2011 [17]	0	1	1	0	1	0	1	1	1	6
Valle 2012-TKA [18]	0	1	1	0	1	1	1	0	1	6
Valle 2012-THA [18]	0	1	1	0	1	1	1	0	1	6
Mraovic 2013 [19]	0	1	1	0	1	1	1	0	1	6
Song 2016-TKA [21]	0	1	1	1	1	1	1	1	1	8
Song 2016-THA [21]	0	1	1	1	1	1	1	1	1	8
Edelstein 2016 [20]	0	1	1	0	1	1	1	1	1	7
Cichos 2018 [22]	0	1	1	0	1	1	1	0	1	6

This Newcastle-Ottawa Scale ranges from 1 to 9 stars and judges the quality of each study regarding the nine domains as listed in the table, with higher scores indicating better study quality

### Association between MetS and VTE after TJA

**Fig. 2:**
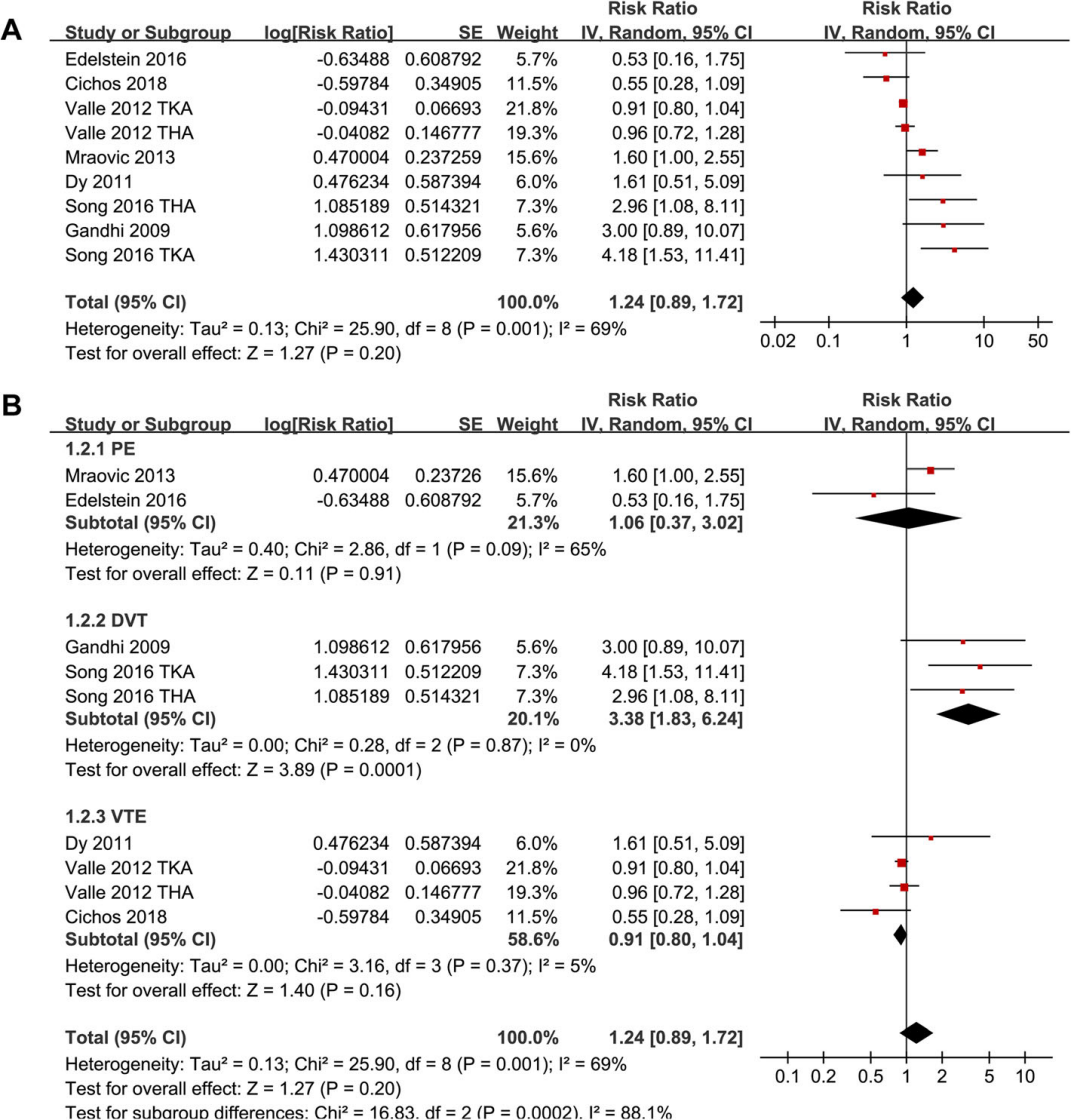
Meta-analysis for the association between MetS and the incidence of VTE after TKA or THA. **a** The main meta-analysis for the overall incidence of VTE. **b** subgroup Analysis according to the categories of VTE events

**Fig. 3:**
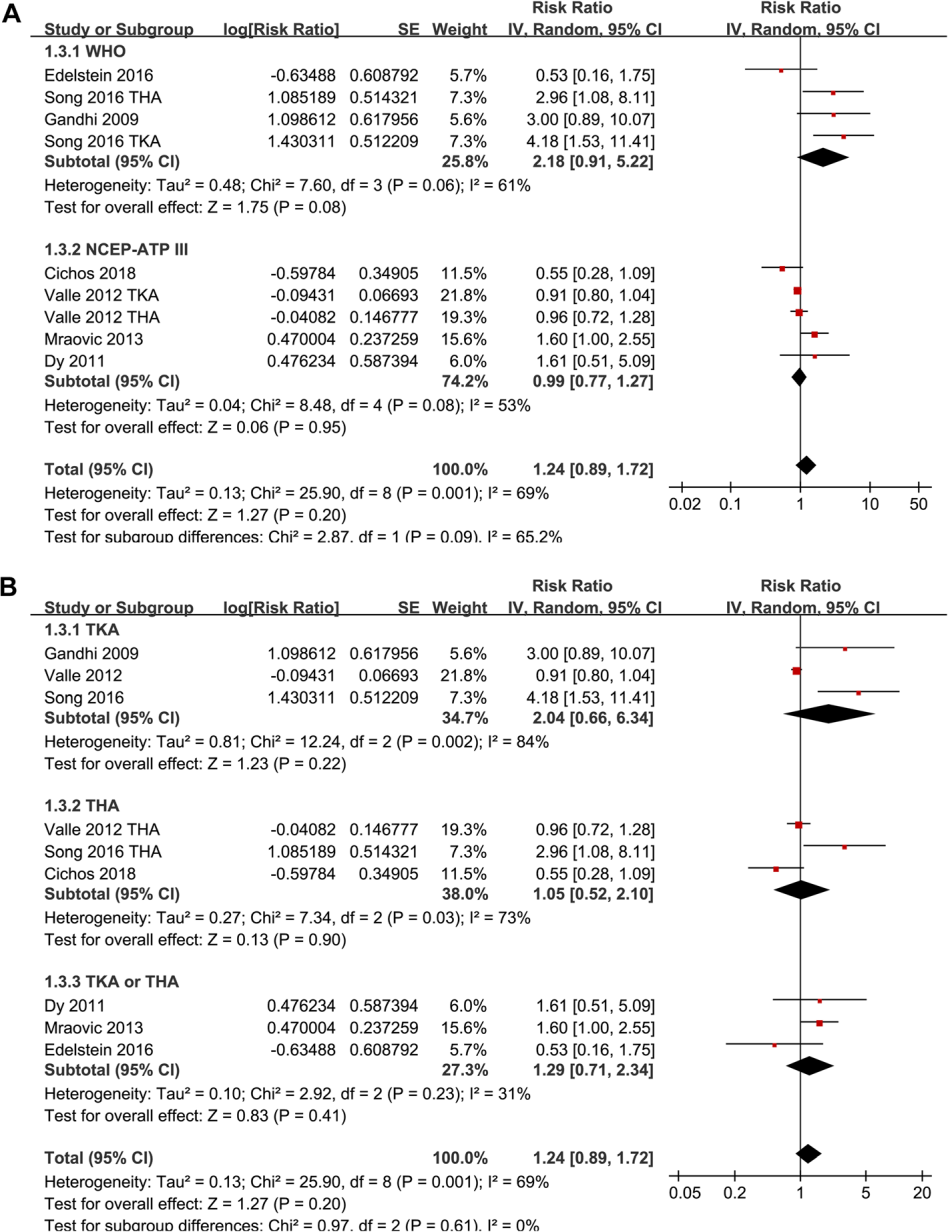
Subgroup analyses the association between MetS and the incidence of VTE after TKA or THA. **a** Subgroup analysis according to the diagnostic criteria of MetS. **b** Subgroup analysis according to the types of surgical procedures

**Fig. 4:**
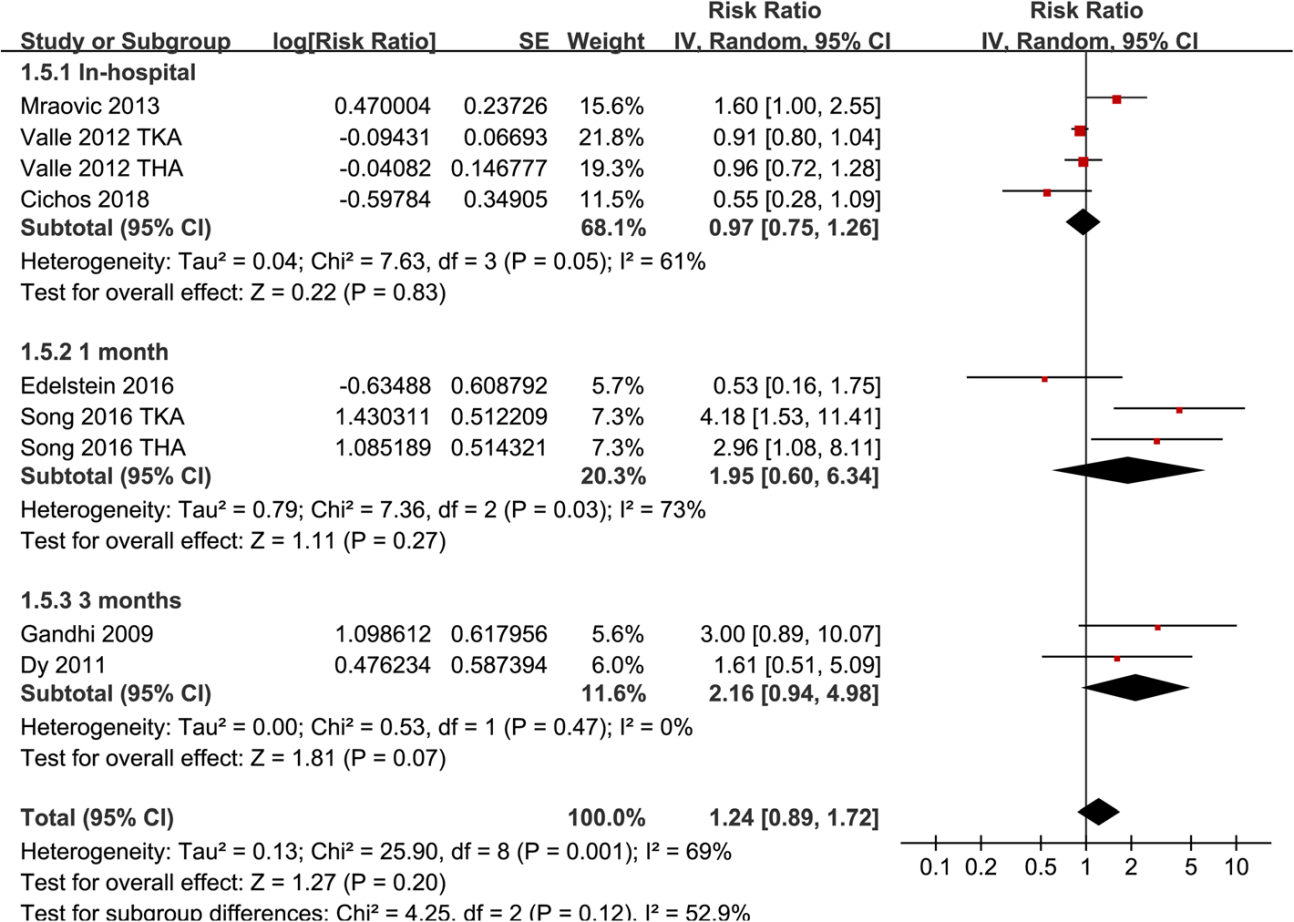
Subgroup analyses the association between MetS and the incidence of VTE after TKA or THA according to the follow-up durations

Significant heterogeneity was detected among the studies that evaluated the association between MetS and VTE risk after TJA (P for Cochrane’s *Q* test = 0.001, *I*^2^ = 69%). Pooled results with a random-effect model showed that MetS was not associated with increased overall VTE after TJA (adjusted RR = 1.24, 95% CI 0.89 ~ 1.72, *p* = 0.20; *I*^2^ = 69%; Fig. 2a). Sensitivity analysis by excluding one study at a time did not significantly affect the results (Table 3). Subgroup analyses showed that MetS was not associated with an increased the risk of PE (RR 1.06, 95% CI 0.37 ~ 3.02, *p* = 0.91) or VTE (RR 0.91, 95% CI 0.80 ~ 1.04, *p* = 0.16), but with an increased risk of DVT after TJA (RR 3.38, 95% CI 1.83 ~ 6.24, *p* < 0.001; *p* for subgroup difference < 0.001; Fig. 2b). Further studies showed that MetS was not significantly associated with an increased risk of VTE after TJA in studies with MetS diagnosed with WHO (RR = 2.18, 95% CI 0.91 ~ 5.22, *p* = 0.08) or NCEP-ATP III criteria (RR = 0.99, 95% CI 0.77 ~ 1.27, *p* = 0.95; Fig. 3a), in studies of patients received TKA (RR = 2.04, 95% CI 0.66 ~ 6.34, *p* = 0.22), THA (RR = 1.05, 95% CI 0.52 ~ 2.10, *p* = 0.90), or both (RR = 1.29, 95% CI 0.71 ~ 2.34, *p* = 0.41; Fig. 3b), and in studies of follow-up within hospitalization (RR = 0.97, 95% CI 0.75 ~ 1.26, *p* = 0.83), 1 month (RR = 1.95, 95% CI 0.60 ~ 6.34, *p* = 0.27), and 3 months (RR = 2.16, 95% CI 0.94 ~ 4.98, *p* = 0.07; Fig. 4) after JTA.

**Table 3:** Results of sensitivity analysis

Studies omitted	RR	95% CI	*I*^2^	*P* for effect
Gandhi 2009 [16]	1.17	0.84 to 1.62	69%	0.35
Dy 2011 [17]	1.22	0.86 to 1.72	72%	0.26
Valle 2012-TKA [18]	1.40	0.89 to 2.22	68%	0.15
Valle 2012-THA [18]	1.38	0.87 to 2.17	73%	0.17
Mraovic 2013 [19]	1.18	0.82 to 1.68	67%	0.37
Song 2016-TKA [21]	1.10	0.82 to 1.48	61%	0.53
Song 2016-THA [21]	1.14	0.83 to 1.58	67%	0.41
Edelstein 2016 [20]	1.31	0.93 to 1.84	72%	0.13
Cichos 2018 [22]	1.37	0.95 to 1.98	70%	0.10

*RR* risk ratio, *CI* confidence interval

### Publication bias

**Fig. 5:**
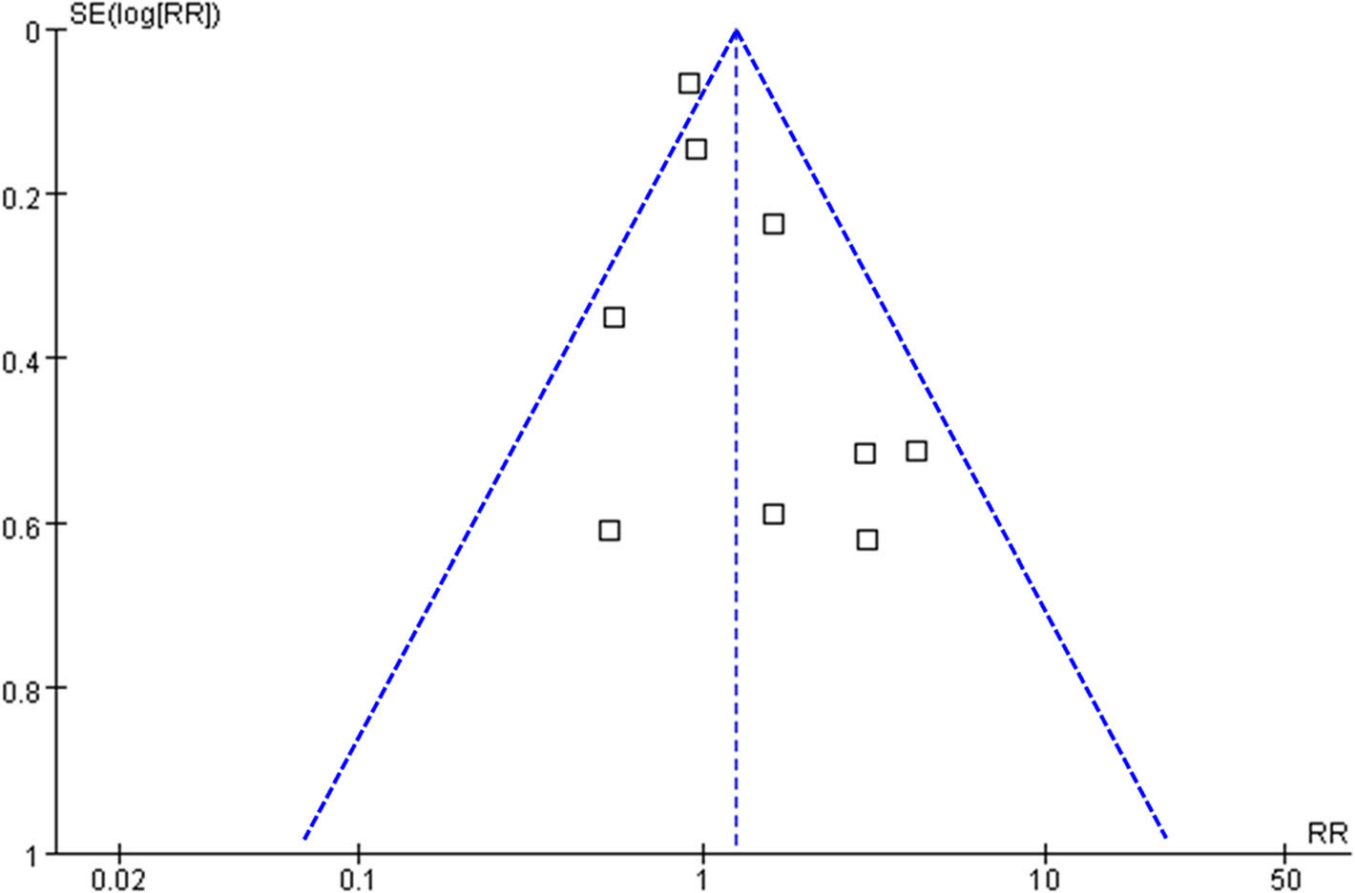
Funnel plots for the meta-analyses of the association between MetS and the incidence of VTE after TKA or THA

The funnel plots for the association between MetS and VTE after TKA or THA were shown in Fig. 5. The plots were symmetrical on visual inspection, suggesting low risks of publication biases. Results of Egger’s regression tests also showed similar results (*p* = 0.389).

## Discussion

In this meta-analysis of cohort study, we found that MetS at baseline was not associated with an increased risk of overall VTE events in patients after TJA. Subgroup analysis showed that MetS was independently associated with an increased risk of DVT, but not PE in patients after TJA. Moreover, the results were not significantly affected by the diagnostic criteria of MetS, the types of surgical procedures, and the follow-up durations. Taken together, current evidence from observational studies suggests that MetS might be associated with an increased risk of DVT, but not PE in patients after TJA.

To the best of our knowledge, our study is the first meta-analysis to evaluate the potential association between MetS and VTE risk in patients after TJA. Although we did not show that MetS was independently associated with an increased incidence of overall VTE, subgroup analysis suggests that MetS was associated with an increased risk of DVT but not PE in these patients. The potential reasons for the findings remain unknown. Generally, PE was resulted by thrombi from the lower extremities of patients with DVT. Due to the routine use of prophylactic measures against VTE in patients after TJA, the incidence of PE in these patients is lower than the incidence of DVT [28]. In our study, the pooled incidence of DVT was 4.7% and compared to 1.6% for PE. It could be hypothesized that potential differences in patient characteristics or diagnostic strategies may be responsible for the observed different associations between MetS with DVT and PE. However, limited datasets included in the subgroup analysis prevented further analysis. Moreover, we found that the results were not significantly affected by the diagnostic criteria of MetS or the types of surgical procedures. Previous studies showed that patients after TKA seem to have a higher incidence of VTE events than those after THA [5, 28]. Although the overall meta-analysis did not show a significant association between MetS and VTE after TJA, subgroup analysis suggested that MetS was associated with increased DVT after TJA. Since only three datasets were included in the subgroup analysis of DVT events, the findings should be validated in large-scale prospective studies. A previous case-control study showed that patients with uncontrolled MetS had a significantly higher incidence of VTE after TJA compared to patients without MetS, while those with controlled MetS had a similar risk of VTE compared to patients without MetS [29], and the VTE events occurred were mainly DVT. These findings may suggest the importance of MetS control for the reducing the risk of DVT after TJA.

The potential pathophysiological mechanisms underlying the association between MetS and increased risk of DVT after TJA may be multifactorial. Patients with MetS are characterized by a persistent activated chronic inflammatory response. Since inflammation is a major activator of coagulation, these patients are generally at hypercoagulative status, thereby vulnerable to venous thrombosis [10, 11]. Moreover, it has been shown that patients with MetS have impaired spontaneous thrombolytic activity as reflected by an impaired expression of tissue-type plasminogen activator [30], which may also be involved in their vulnerability to thrombotic events. Besides, patients with MetS tend to be overnutrition and are more likely to be exposed to high-fat diet. Previous studies showed that high-fat diet maintains high endogenous thrombin potential, which is associated with venous and arterial thrombosis independently of obesity and insulin resistance [31]. However, the correlation of nutritional status and VTE risk could be complex, since recent studies showed that the poor nutrition status was also associated with a high risk of developing DVT, particularly for those who underwent major surgeries [32, 33]. Further studies are needed to elucidate the mechanisms underlying the association between MetS and DVT.

Our study has limitations, which should be considered when interpreting the results. Firstly, as a meta-analysis of observational studies, although we combined RR data after adjustment of potential confounding factors, we could not exclude other residual factors that may confound the association between MetS and DVT after TJA, such as the comorbidities of the patients and concurrent medications [34]. Secondly, due to the limited number of the included studies, the result of the subgroup analysis should be interpreted with caution. In addition, most of the included studies were retrospective, which may be limited by the recall bias compared to prospective studies. Moreover, the lengths of follow-up varied among the included studies. However, subgroup analysis showed that MetS was not associated with increased VTE risk after TJA in studies of follow-up within hospitalization, 1 month, and 3 months after JTA. Besides, the long-term (> 3 months) association between MetS and VTE risk after TJA remains to be determined in future studies. Finally, a causative relationship between MetS and increased DVT after TJA should not be retrieved from our results since it is a meta-analysis of observational studies.

## Conclusions

In conclusion, our meta-analysis showed that current evidence from observational studies suggests MetS might increase the risk of DVT but not PE in patients that received TJA. Although these findings should be validated in prospective studies, the results of this meta-analysis may suggest the importance of MetS control for reducing the risk of DVT after TJA.

## Data Availability

The raw data used in our study are included in the manuscript with tables, figures, and its supplementary information files. All the authors agreed that the data could be shared if required by the researchers.

## Abbreviations

MetS: Metabolic syndrome
VTE: Venous thromboembolism
DVT: Deep vein thrombosis
PE: Pulmonary embolism
TJA: Total joint arthroplasty
TKA: Total knee arthroplasty
THA: Total hip arthroplasty
WHO: World Health Organization
NCEP-ATP III: Revised National Cholesterol Education Program’s Adults Treatment Panel III
MOOSE: Meta-analysis of Observational Studies in Epidemiology
RRs: Risk ratios
SE: Stand error
